# Physiological evidence for plasticity in glycolate/glycerate transport during photorespiration

**DOI:** 10.1007/s11120-016-0277-3

**Published:** 2016-06-01

**Authors:** Berkley J. Walker, Paul F. South, Donald R. Ort

**Affiliations:** Global Change and Photosynthesis Research Unit, United State Department of Agriculture/Agricultural Research Services, Urbana, IL 61801 USA; Carl R. Woese Institute for Genomic Biology, University of Illinois, Urbana, IL 61801 USA; Department of Plant Biology, University of Illinois, Urbana, IL 61801 USA; Institute of Plant Biochemistry, Cluster of Excellence on Plant Sciences, Heinrich Heine University, 40225 Düsseldorf, Germany

**Keywords:** Photorespiration, Photosynthesis, Quantum efficiency, Abiotic stress, Central metabolism, Photosynthetic models

## Abstract

Photorespiration recycles fixed carbon following the oxygenation reaction of Ribulose, 1–5, carboxylase oxygenase (Rubisco). The recycling of photorespiratory C2 to C3 intermediates is not perfectly efficient and reduces photosynthesis in C3 plants. Recently, a plastidic glycolate/glycerate transporter (PLGG1) in photorespiration was identified in *Arabidopsis thaliana*, but it is not known how critical this transporter is for maintaining photorespiratory efficiency. We examined a mutant deficient in PLGG1 (*plgg1*-*1*) using modeling, gas exchange, and Rubisco biochemistry. Under low light (under 65 μmol m^−2^ s^−1^ PAR), there was no difference in the quantum efficiency of CO_2_ assimilation or in the photorespiratory CO_2_ compensation point of *plgg1*-*1*, indicating that photorespiration proceeded with wild-type efficiency under sub-saturating light irradiances. Under saturating light irradiance (1200 μmol m^−2^ s^−1^ PAR), *plgg1*-*1* showed decreased CO_2_ assimilation that was explained by decreases in the maximum rate of Rubisco carboxylation and photosynthetic linear electron transport. Decreased rates of Rubisco carboxylation resulted from probable decreases in the Rubisco activation state. These results suggest that glycolate/glycerate transport during photorespiration can proceed in moderate rates through an alternative transport process with wild-type efficiencies. These findings also suggest that decreases in net CO_2_ assimilation that occur due to disruption to photorespiration can occur by decreases in Rubisco activity and not necessarily decreases in the recycling efficiency of photorespiration.

**Electronic supplementary material:**

The online version of this article (doi:10.1007/s11120-016-0277-3) contains supplementary material, which is available to authorized users.

## Introduction

Gross CO_2_ assimilation during photosynthesis is diminished by CO_2_ loss from photorespiration in C3 plants. This CO_2_ loss is dependent on temperature and CO_2_ partial pressures and comprises the largest single loss of carbon to an illuminated C3 leaf, resulting in an annual decrease of 322 trillion dietary calories in the Midwestern United States alone (Walker et al. 2016b; Sharkey 1988). Photorespiration is initiated when Ribulose 1–5 bisphosphate carboxylase oxygenase (Rubisco) reacts with O_2_ instead of CO_2_ and produces 3-phosphoglycerate and 2-phosphoglycolate. Photorespiration recycles phosphoglycolate into the C3 intermediate 3-phosphoglycerate in reactions involving the chloroplast, peroxisome, and mitochondrion (Foyer et al. 2009; Bauwe et al. 2010). According to current understanding, this photorespiratory recycling is not perfectly efficient on a carbon basis in that for every four carbons that enter photorespiration as two phosphoglycolate, one is lost as CO_2_, while the remaining three form 3-phosphoglycerate.

There is evidence that photorespiratory carbon efficiency can decrease further when photorespiration is disrupted genetically. Photorespiratory mutants typically have decreased net CO_2_ assimilation rates, which could be driven by decreases to photorespiratory efficiency (Somerville and Ogren 1979, 1980a, b; Timm et al. 2012; Pick et al. 2013). Decreases in photorespiratory efficiency increase the stoichiometry of CO_2_ release per Rubisco oxygenation (α) above the normally assumed value of 0.5. ^18^O_2_ and ^16^O_2_ exchange experiments indicate that α increases in *Arabidopsis thaliana* missing the photorespiratory genes responsible for forming glycerate, which is then transported in the chloroplast for incorporation into the C3 cycle (hydroxypyruvate reductase (*hpr*) and peroxisomal malate dehydrogenase (*pmdh1pmdh2*), Cousins et al. 2008, 2011). Decreases in photorespiratory efficiency in *hpr, pmdh1pmdh2,* and *hprpmdh1pmdh2* suggest the presence of alternative metabolic fates for downstream products of glycolate metabolism and highlight the plasticity of photorespiration. For this work, we define plasticity as the ability for photorespiration to channel metabolites through pathways or protein-mediated processes (such as transport) that are not currently included in dogmatic portrayal of photorespiration. While this decrease in photorespiratory recycling efficiency has been characterized explicitly for mutants deficient in hydroxypyruvate reduction, it has not yet been examined in other photorespiratory mutants.

In this work, we have defined decreases in photorespiratory efficiency as increases in the amount of CO_2_ released per Rubisco oxygenation (α), although we recognize it could be also considered on an energy basis. Decreases in photorespiratory efficiency are reflected in increases to the CO_2_ compensation (Γ) and photorespiratory CO_2_ compensation point (Γ^*^), which scale with α according to the relationship.

1 $$\varGamma^{*} = \frac{\alpha O}{{S_{\text{c/o}} }}$$

and

2 $$\varGamma = \frac{{\varGamma^{*} + K_{\text{c}} \left( {1 + O/K_{\text{o}} } \right)R_{\text{d}} /V_{{{\text{c}}\hbox{max} }} }}{{1 - R_{\text{d}} /V_{{{\text{c}}\hbox{max} }} }},$$

where *O*, *S*_c/o_, *K*_c_, *K*_o_, *R*_d_, and *V*_cmax_ represent the partial pressure of oxygen, specificity of Rubisco for reaction with CO_2_ relative to oxygen, *K*_m_ for CO_2_, *K*_m_ for oxygen, rates of day respiration, and the maximum rate of Rubisco carboxylation, respectively (von Caemmerer 2000; Cousins et al. 2008, 2011; Timm et al. 2008, 2012). Decreases in photorespiratory efficiency should also decrease the quantum efficiency of net CO_2_ assimilation ($\varPhi_{{{\text{CO}}_{2} }}$), since more CO_2_ would be lost through photorespiration per photon of light absorbed; however, we are unaware of any modeling framework quantifying the impact of changes in α on $\varPhi_{{{\text{CO}}_{2} }}$.

Somewhat recently a key photorespiratory plastidic glycolate glycerate antiporter gene (*PLGG1*) was identified (Pick et al. 2013), over 30 years after the transporter had been extensively characterized biochemically (Howitz and McCarty 1982, 1986, 1991; Howitz and McCarty 1985). *PLGG1* is present in only one copy in *Arabidopsis thaliana* (*plgg1*) and is thought to be the key plastidic transporter of photorespiratory carbon skeletons by exchanging glycolate generated following the oxidation by Rubisco for glycerate produced in the peroxisome. *plgg1*-*1* shows several hallmarks of a photorespiratory mutant such as visible leaf damage following 7 days transition to ambient from high CO_2_ growth conditions and decreased net CO_2_ assimilation at ambient compared to high CO_2_; however, the visual phenotype is not as severe when compared side-by-side with a mutant lacking serine hydroxymethyltransferase (Pick et al. 2013, supporting Fig. 5 therein).

It is also interesting that given its central role to photorespiration and the presence of only a single copy in *A. thaliana*, *plgg1* managed to evade discovery despite several decades of photorespiratory screens using a variety of detection methods (Somerville 1986; Badger et al. 2009; Timm and Bauwe 2013). Additionally, while the biochemical characterization of PLGG1 reveals it to be an antiporter of glycolate and glycerate with a 1:1 stoichiometry, the stoichiometry would need to be 2:1 to explain carbon transport in the current schema of photorespiration (Howitz and McCarty 1982, 1986, 1991; Ogren 1984; Howitz and McCarty 1985). That PLGG1 evaded detection for so long could have been the result plasticity in glycolate/glycerate exchange across the chloroplast envelope membrane and/or plasticity within the photorespiratory metabolic pathway itself (Timm et al. 2012).

The purpose of this study is to investigate the plasticity of glycolate/glycerate transport during photorespiration through measurements of gas exchange of *plgg1*-*1* under photorespiratory and non-photorespiratory conditions. We also developed a model to determine the impact of increases in α to $\varPhi_{{{\text{CO}}_{2} }}$ and compared this model to measurements under photorespiratory and non-photorespiratory conditions. We parameterize this model for *plgg1*-*1* based on the α value from *hprpmdh1pmdh2* hypothesizing that a full blockage of glycerate return to the chloroplast would have a similar metabolic phenotype as a mutant defective in the immediately downstream reaction forming the glycerate, assuming that chloroplastic export of glycolate can occur via simple diffusion. This work revealed that PLGG1 does not appear essential to maintain photorespiratory efficiency on a CO_2_ exchange basis under low irradiance (under 65 μmol m^−2^ s^−1^ PAR) and that decreases in net CO_2_ assimilation instead are driven mainly by decreases in the activation state of Rubisco and capacity for electron transport. These findings indicate that photorespiration is plastic in transport processes and suggest a mechanism for the regulation of photosynthesis by photorespiration.

## Materials and methods

### Growth conditions and transgenic confirmation

Seeds for *pgg1*-*1* (SALK line SALK_053469C) were obtained from the Arabidopsis Biological Resource Center. T-DNA insertional interruption and homozygosity were confirmed by PCR on *plgg1*-*1* using the primers and methods reported previously (Pick et al. 2013). Wild-type *Arabidopsis thaliana* (Col-0) and *plgg1*-*1* were stratified in distilled water for 2–3 days at 4 °C and sown directly on soil. Plants were grown in a climate-controlled cabinet (Conviron, Winnipeg, Manitoba, Canada) with day/night cycles of 8/16 h and 23/18 °C under an irradiance of 250 μmol m^−2^ s^−1^. CO_2_ was maintained at ~200 Pa and periodically monitored using an infra-red gas analyzer (SBA-5, PP systems, Amesbury, MA, USA) and datalogger (CR1000, Campbell Scientific, Logan, UT, USA). Identical chambers with no CO_2_ enrichment (~40 Pa) were used for ambient treatments. Plants were watered as needed and fertilized weekly (Peters 20-20-20, J.R. Peters, Allentown, PA, USA).

### Gas exchange and leaf-level chlorophyll fluorescence

The youngest fully expanded leaves of 30–40 days old plants were used for gas exchange and subsequent analysis. The plants where measured during the end of the principle growth stage 3 (Boyes et al. 2001) and the youngest fully expanded leaf was defined as the youngest leaf that had begun petiolar elongation and was expanded to an area larger than ~3 cm^2^. Gas exchange was performed using a LI-COR 6400 XT with 2 cm^2^ fluorescence measuring head (LI-COR Biosciences, Lincoln, NE, USA) with gasket leaks corrected as outlined in the manual. The multiphase flash protocol was employed for leaf-level chlorophyll fluorescence with appropriate optimizations of flash intensity and kinetics (Loriaux et al. 2013). The operational quantum efficiency of PSII ($\varPhi_{\text{PSII}}$), a unitless indicator of the proportion of light energy absorbed by PS II that is put towards plastoquinone reduction, was determined according to standard PAM fluorescence equations (Genty et al. 1989). Chloroplastic CO_2_ was determined from intercellular CO_2_ assuming a mesophyll conductance of 3 mol m^−2^ s^−1^ MPa^−1^ as determined previously in Arabidopsis grown under elevated CO_2_ and similar conditions (Walker et al. 2013).

Light response curves were measured by acclimating a clamped leaf under 1200 μmol m^−2^ s^−1^ PAR and then decreasing the irradiance stepwise to 380, 120, 65, 40, 30, 27, 18, and 10 μmol m^−2^ s^−1^ at both high and low intercellular CO_2_ (~10 and 100 Pa with 21 kPa oxygen) and low oxygen (2 kPa and ~25 Pa intercellular CO_2_). Following the light response curve, leaf absorbance was determined using an integrating sphere (Jaz Spectroclip, Ocean Optics, Dunedin, FL, USA) and used to determine absorbed irradiance. The quantum efficiency of net CO_2_ fixation ($\varPhi_{{{\text{CO}}_{2} }}$) was determined as the slope of the response of net CO_2_ assimilation to increasing absorbed irradiance up to 30 μmol m^−2^ s^−1^. Low oxygen (2 kPa) was provided using mass flow controllers regulating oxygen and nitrogen flow using a custom-built Raspberry-Pi controller.

The photorespiratory CO_2_ compensation point (Γ^*^) and R_d_ were determined from the common intersection of the linear portions of photosynthetic CO_2_ response curves measured at sub-saturating irradiances using slope-intercept regression and assuming a single mesophyll conductance (Laisk 1977; von Caemmerer et al. 1994; Walker and Ort 2015; Walker et al. 2016a). CO_2_ assimilation was measured stepwise from 10 to 1.5 Pa CO_2_ using a LI-COR 6400 XT modified to reach low CO_2_ concentrations at irradiances of 250, 150, 75, and 50 μmol m^−2^ s^−1^. Standard errors on all Γ^*^ measurements where smaller when slope-intercept regression was applied compared to standard common intercept averaging (Walker et al. 2016a).

Full photosynthetic CO_2_ response curves were measured using CO_2_ partial pressures in the following order: 40, 25, 15, 5, 10, 40, 120, 200, 160, 80, and 40 Pa CO_2_, under saturating irradiance (1200 μmol m^−2^ s^−1^) and fitted to determine *V*_cmax_ and *J*_max_ using standard biochemical models of photosynthesis and Arabidopsis-specific Rubisco kinetics (Sharkey et al. 2007; von Caemmerer and Farquhar 1981; Walker et al. 2013).

### Modeling the quantum efficiency of CO_2_ fixation and compensation point

To determine the modeled impact of an increase to the CO_2_ release per Rubisco oxygenation (α), $\varPhi_{{{\text{CO}}_{2} }}$ is defined as

3 $$\varPhi_{{{\text{CO}}_{2} }} = \frac{\text{GA}}{{{\text{PAR}}_{\text{abs}} }},$$

where GA and PAR_abs_ represent gross CO_2_ assimilation and absorbed irradiance. According to the standard model for leaf photosynthesis, GA accounting for CO_2_ fixation and photorespired loss is represented by

4 $${\text{GA}} = V_{\text{c}} - \alpha V_{\text{o}},$$

where *V*_c_ and *V*_o_ are rates of Rubisco carboxylation and oxygenation and *V*_o_/*V*_c_ is determined as

5 $$\frac{{V_{\text{o}} }}{{V_{\text{c}} }} = \frac{O}{{CS_{\text{c/o}} }},$$

where *O*, *C*, and *S*_c/o_ represent the oxygen partial pressure, CO_2_ partial pressure, and Rubisco specificity (von Caemmerer 2000). By re-arrangement of Eqs. 4 and 5,

6 $${\text{GA}} = V_{\text{c}} \left( {1 - \frac{\alpha O}{{CS_{\text{c/o}} }}} \right).$$

To model the relationship between PAR_abs_ and GA, assume that the rate of NADPH produced from photochemistry (*V*_NADPH_) is defined as

7 $$2V_{\text{NADPH}} = 0.5*{\text{PAR}}_{\text{abs}} *\phi_{\text{PSII}}$$

and

8 $$V_{\text{NADPH}} = 2V_{\text{c}} + 2V_{\text{o}}$$

according to Ruuska et al. (2000a). By combining Eqs. 5, 7, and 8,

9 $${\text{PAR}}_{\text{abs}} = \frac{{4V_{\text{c}} \left( {2 + \frac{2O}{{CS_{\text{c/o}} }}} \right)}}{{\phi_{\text{PSII}} }}$$

which is combined with Eqs. 3 and 6 to produce

10 $$\varPhi_{{{\text{CO}}_{2} }} = \frac{{\phi_{\text{PSII}} \left( {CS_{\text{c/o}} - \alpha O} \right)}}{{8\left( {CS_{\text{c/o}} + O} \right)}}.$$

Equation 10 modeled $\varPhi_{{{\text{CO}}_{2} }}$ using the average $\varPhi_{\text{PSII}}$ values from the lowest irradiances of the measured light response curves for each CO_2_ as presented in Table 3 and an assumed S_c/o_ of 2599 calculated on a partial pressure basis. This model provides a novel framework to examine the impact of changes in photorespiratory efficiency to net CO_2_ gas exchange. It is interesting to note that rates of day respiration (*R*_d_) are not needed when measuring or modeling the $\varPhi_{{{\text{CO}}_{2} }}$ as long as R_d_ is assumed constant. This is because $\varPhi_{{{\text{CO}}_{2} }}$ is defined as the increase of net CO_2_ fixation per quantum of light absorbed or the initial slope of a light response curve. Changes in R_d_ only impact the y-intercept of this relationship and not the slope.

### Response of *plgg1*-*1* to ambient CO_2_

Wild type and *plgg1*-*1* were removed from high CO_2_ growth conditions and dark adapted for at least 20 min before measurement of *F*_v_/*F*_m_ in a chlorophyll fluorescence imager (CF imager, Technologica Ltd, Colchester, UK). Using this image, youngest fully expanded leaves with homogenous *F*_v_/*F*_m_ values were selected for steady-state gas exchange at 40 Pa CO_2_. The measurement was repeated on the same plants following 2, 4, and 6 days at ambient CO_2_.

### Rubisco content, activation state, protein content, and chlorophyll content of *plgg1*-*1*

Rubisco binding sites were determined on flash frozen leaf disks extracted immediately following photosynthetic CO_2_ response curve measurements (Walker et al. 2013). Following the last CO_2_ response curve measurement at 40 Pa CO_2_ and 1200 μmol m^−2^ s^−1^ PAR, an ~1.5 cm^2^ leaf punch was made from the leaf from where it had been enclosed in the gas exchange chamber. The leaf disk was transferred into a micro-centrifuge tube and dropped into a container of liquid nitrogen. This process took less than 3 s for each sample. Following storage at −80 °C, frozen disks were disrupted in a glass homogenizer in ice cold buffer (50 mM HEPES–NaOH (pH 7.8), 1 % polyvinylpolypyrrolidone, 1 mM EDTA, 10 mM DTT, 0.1 % Triton, and 1X Sigma protease inhibitor cocktail), centrifuged at 17,000*×g* relative centrifugal force for 5 min at 4 °C and activated in 15 mM MgCl_2_ and 15 mM NaHCO_3_ for 30 min at room temperature and placed on ice. Rubisco content was determined from the stoichiometric binding of radiolabeled ^14^C-carboxy-arabinitol-bisphosphate (^14^CABP) (Ruuska et al. 1998). Protein content was determined using the Bradford method (Bio-Rad Protein Assay, Bio-Rad, Hercules, CA, USA).

Activation state was determined in a separate set of plants flash frozen following 20-min acclimation in conditions identical to the A–Ci curve measurements by measuring initial and chemically activated Rubisco activity in raw extracts. Initial Rubisco activity was assayed following rapid extraction at 4 °C (50 mM HEPES–NaOH, pH 7.8, 1 % polyvinylpolypyrrolidone, 1 mM EDTA, 10 mM DTT, 5 mM MgCl2, 0.1 % Triton, and 1X Sigma-Aldrich plant protease inhibitor cocktail) using a 2-mL tissue grinder. Following homogenization, the extract was centrifuged at 21,100×*g* for 20 s at 4 °C and the supernatant assayed for initial activity spectrophotometrically from the enzymatically coupled conversion of NADH to NAD + (100 mM EPPS-NaOH, pH 8.0, 10 mM MgCl2, 1 mM EDTA, 1 mM ATP, 5 mM phosphocreatine, 20 mM NaHCO3, 0.2 mM NADH, and 0.5 mM RuBP with coupling enzymes: 25 U mL^−1^ creatine phosphokinase, 250 U mL^−1^ carbonic anhydrase, 25 U mL^−1^ 3-phosphoglycerate kinase, 20 U mL^−1^ glyceraldehyde-3-phosphate dehydrogenase, 20 U mL^−1^ glycerol-3-phosphate dehydrogenase, and at least 55 U mL^−1^ triosephosphate isomerase) (Ruuska et al. 2000; Yamori and von Caemmerer 2009; Carmo-Silva and Salvucci 2013; Kim et al. 2016). The assay was optimized so that initial activity was measured within 2 min of removal of the disks from liquid nitrogen to minimize changes in activation state. Final activity was determined following 10 min of activation assay buffer and activation state determined as the ratio of initial to final activity. All assays and incubations were performed at 25 °C. In initial optimizations, maximum Rubisco activity of extracts was found following 8–13 min of activation. Initial rates were determined using linear regression of the first minute following reaction initiation.

### Statistics

**Fig. 1:**
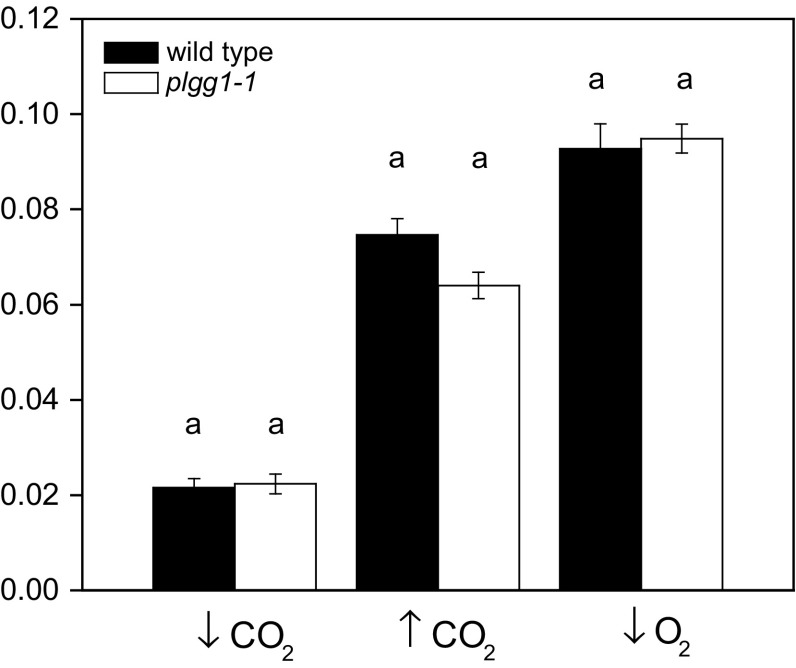
Light response curves of wild type and *plgg1*-*1*. The quantum efficiency of CO_2_ fixation ($\varPhi_{{{\text{CO}}_{2} }}$) was measured on each plant under elevated (90 Pa), low (10 Pa) intercellular CO_2_ partial pressures, and ambient CO_2_ with low (2 kPa) oxygen. Means of *n* = 5–8 are shown with ± standard error. Significant differences within a measuring condition are indicated with different letters according to a Student's *t* test with *p* < 0.05

A Student's *t* test (*p* < 0.05) was used for comparisons between *plgg1*-*1* and wild type when only one treatment and time where compared (Tables 2, 4; Fig. 1). A two-way (genotype by day) repeated measures ANOVA was used to test significance (*p* < 0.05) of experiments involving the same plants with multiple sampling times (Fig. 3). A one-way ANOVA was used to test remaining significance (*p* < 0.05). All ANOVA were followed with a Tukey’s post hoc test and determined using statistical software (OriginPro 9.0, OriginLab, Northhampton, MA, USA).

## Results

### Decreases in photorespiratory efficiency decrease modeled $\varPhi_{{{\text{CO}}_{2} }}$ under photorespiratory conditions

A modeling approach was used to predict the impact of increases in α on $\varPhi_{{{\text{CO}}_{2} }}$ under high (10 Pa CO_2_ and 20 kPa O_2_) and low photorespiratory conditions (90 Pa CO_2_ and 20 kPa O_2_, 25 Pa CO_2_ and 2 kPa O_2_, Table 1). Values of α were assumed to be 0.5 for wild-type photorespiration as predicted from the dogmatic scheme of photorespiration and 0.8 based on previous analysis of *hprpmdh1pmdh2*, which showed increases in α (von Caemmerer 2000; Cousins et al. 2008, 2011). This simulation revealed that the decreases in photorespiratory efficiency of carbon recycling found in *hprpmdh1pmdh2* would be expected to decrease $\varPhi_{{{\text{CO}}_{2} }}$ by ~30 % under photorespiratory conditions (10 Pa CO_2_ and 20 kPa O_2_) but have little impact on $\varPhi_{{{\text{CO}}_{2} }}$ when photorespiration is suppressed by high CO_2_ (3 % decrease) or low O_2_ (no difference). The high and low photorespiratory conditions in the model produced meaningful controls for comparison to measurements since the predictions produce situations where α is and is not expected to impact $\varPhi_{{{\text{CO}}_{2} }}$. These modeled values were next compared to measured observations to determine if there was support for an increase in α by examining $\varPhi_{{{\text{CO}}_{2} }}$.

**Table 1:** Modeled impact of changes in CO_2_ release per Rubisco oxygenation on maximum quantum efficiency of CO_2_ fixation

*C*_c_ (Pa)	O_2_ (kPa)	*V*_o_/*V*_c_	$\varPhi_{\text{PSII}}$	*α*	$\varPhi_{{{\text{CO}}_{2} }}$ × 100
10	20	0.769	0.72	0.50	3.1
10	20	0.769	0.72	0.80	2.0
90	20	0.085	0.66	0.50	7.3
90	20	0.085	0.66	0.80	7.1
25	2	0.031	0.67	0.50	7.9
25	2	0.031	0.67	0.80	7.9

The maximum quantum efficiency of CO_2_ fixation ($\varPhi_{{{\text{CO}}_{2} }}$) was modeled in response to changes in the amount of CO_2_ released per Rubisco oxygenation (*α*). The chloroplastic CO_2_ partial pressure (*C*_c_) and quantum efficiency of PSII ($\varPhi_{\text{PSII}}$) values used were averaged from the $\varPhi_{{{\text{CO}}_{2} }}$ measurements presented in Fig. 1. Rubisco specificity was assumed to be 2888 Pa/Pa as determined from wild-type Γ^*^ measurements from Table 2 and Eq. 2

### Measurements of Γ^*^ and $\varPhi_{{{\text{CO}}_{2} }}$ reveal no large change in photorespiratory recycling efficiency in *plgg1*-*1*

We next measured Γ^*^ to determine if there was gas exchange evidence for decreases in the efficiency of carbon recycling during photorespiration in *plgg1*-*1* to compared to modeled values (Table 2). While *plgg1*-*1* had a larger Γ^*^ (~25 %), this difference was not quite significant as determined by a Student's *t* test (*p* = 0.06). Rates of day respiration (*R*_d_) where identical between genotypes, but Γ was significantly larger in *plgg1*-*1*.

**Table 2:** The photorespiratory CO_2_ compensation point of wild type and plgg1-1

	Wild type	*plgg1*-*1*
Γ^*^ (Pa)	3.6 ± 0.3^a^	4.5 ± 0.1^a^
Γ (Pa)	4.6 ± 0.1^a^	5.9 ± 0.1^b^
*R*_d_ (μmol m^−2^ s^−1^)	0.5 ± 0.0^a^	0.6 ± 0.0^a^

Measurements of the photorespiratory CO_2_ compensation point (Γ^*^), CO_2_ compensation point (Γ), and day respiration (*R*_d_) where made on wild type and *plgg1*-*1* using slope-intercept regression on CO_2_ response curves measured under sub-saturating irradiances. The presented Γ values are from measurements made at 1200 PAR. Means of *n* = 3–4 are shown with ± standard error. Significant differences within a measurement are indicated with different letters according to a Student's *t* test with *p* < 0.05

To test the carbon recycling efficiency of impaired glycerate/glycolate transport in photorespiration, $\varPhi_{{{\text{CO}}_{2} }}$ was determined from light response curves measured in *plgg1*-*1* under high photorespiratory conditions (10 Pa CO_2_ and 20 kPa O_2_) and low photorespiratory conditions (90 Pa CO_2_ and 20 kPa O_2_, 2 kPa O_2_ and 25 Pa CO_2_, Fig. 1). As expected, $\varPhi_{{{\text{CO}}_{2} }}$ was lower under low CO_2_ and increased under high CO_2_ generally. There was no statistical difference between *plgg1*-*1* and wild type under conditions of low CO_2_, neither were differences found under high CO_2_. The absolute values of $\varPhi_{{{\text{CO}}_{2} }}$ agreed well with our modeled values that assumed α was equal to 0.5 under low and high CO_2_. This suggests that photorespiration was able to maintain efficiency despite genetic disruption in *plgg1*-*1* under conditions of high photorespiration relative to CO_2_ assimilation (low CO_2_).

**Fig. 2:**
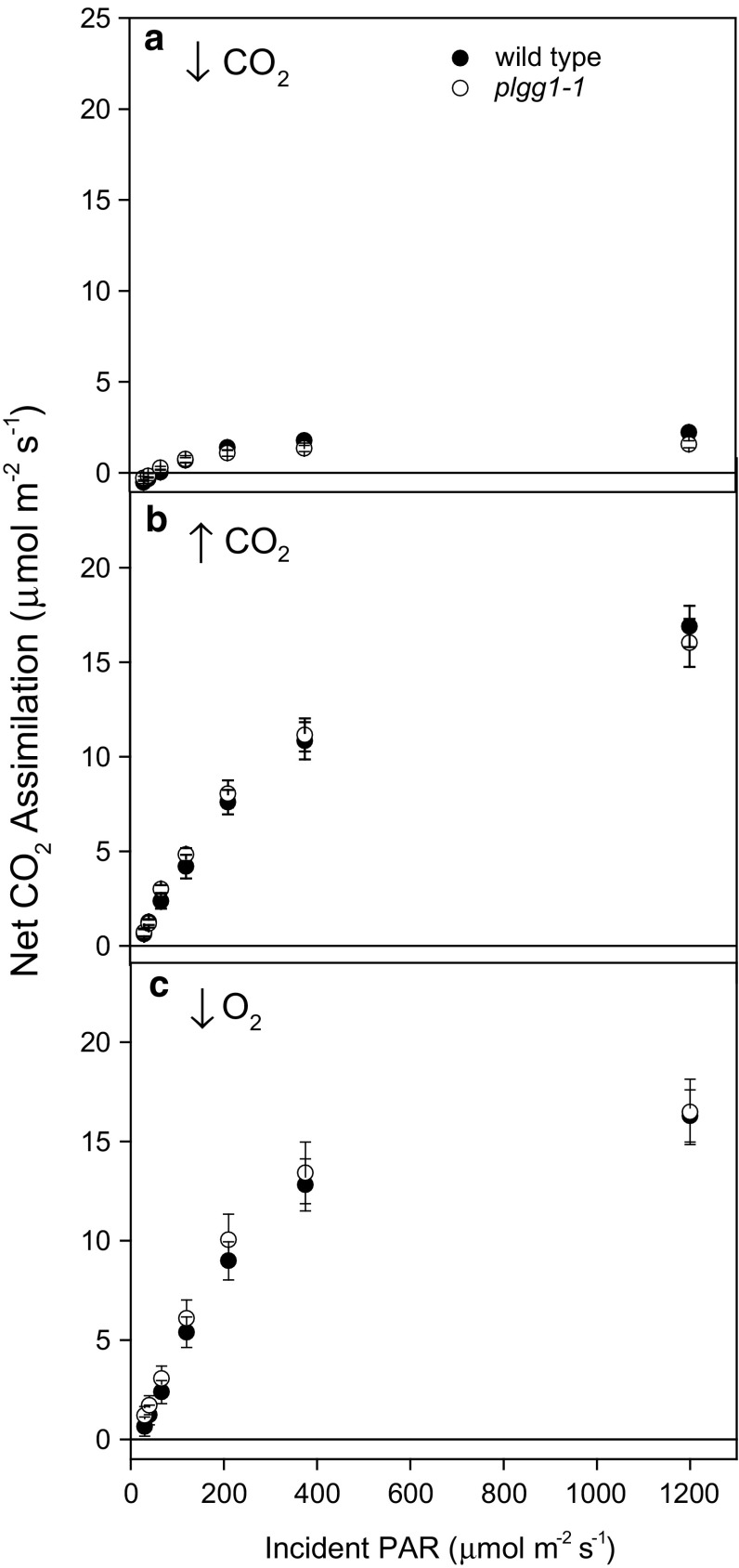
The response of net CO_2_ assimilation to various irradiances under high (90 Pa, **a**), low (10 Pa, **b**) CO_2_ partial pressures, or low oxygen (2 kPa, **c**). Ambient CO_2_ was adjusted to give C_i_ values similar to those used for modeling in Table 1 and low O_2_ was set using mass flow controllers. Measurements were made during a light response curve at each CO_2_ treatment on wild type and *plgg1*-*1*. *Bars* represent means of *n* = 5 with ± standard error

Since $\varPhi_{{{\text{CO}}_{2} }}$ is measured under very low irradiances (<100 μmol m^−2^ s^−1^) from the initial close-to-linear portion of the light response curve, we next examined the full light response curve to understand the impact of disruption of PLGG1 to net photosynthesis under increasing irradiances and gross photorespiratory flux. The full light response curves from these measurements reveal that wild type and *plgg1*-*1* show similar responses to increasing irradiances when photorespiration is suppressed through high CO_2_ or low O_2_ (Fig. 2). *plgg1*-*1* shows a decrease in net photosynthetic CO_2_ assimilation under photorespiratory conditions.

### CO_2_ response curves reveal that decreased assimilation in *plgg1*-*1* results from decreased photosynthetic biochemistry

The response of net CO_2_ assimilation to CO_2_ (A–C_i_ curves) was measured under saturating irradiance to determine at what C_i_*plgg1*-*1* starts showing decreased photosynthetic rates under constant illumination as the rate of photorespiration is varied. A–C_i_ curves where measured on plants taken directly from a high CO_2_ growth condition (~200 Pa, day 0) and following 48 h under ambient CO_2_ (~40 Pa, day 2). On day 0 with measurement CO_2_ concentrations close to ambient (40 Pa), *plgg1*-*1* showed a ~40 % decrease in assimilation compared to wild type (Table 3, S2). High CO_2_ (200 Pa) partially rescued *plgg1*-*1* (to 30 % of wild type). Photosynthetic biochemical parameters derived from these A–C_i_ curves were lower in *plgg1*-*1*. The maximum rate of Rubisco carboxylation (*V*_cmax_) was reduced by 60 % and maximum rates of electron transport (*J*_max_) was reduced by 30 % in the null mutant. These decreases in *plgg1*-*1* became more severe following 2 days growth at ambient CO_2_. Assimilation rates at 40 and 200 Pa CO_2_ decreased to 60 and 50 % of wild type, while *V*_cmax_ and *J*_max_ decreased by 70 and 50 % of wild type.

**Table 3:** Biochemical characteristics of wild type and plgg-1

	Day 0		Day 2	
	Wild type	*plgg1*-*1*	Wild type	*plgg1*-*1*
A_40_ (μmol m^−2^ s^−1^)	13.0 ± 0.8^a^	7.6 ± 1.0^b^	13.3 ± 0.6^a^	5.5 ± 0.6^b^
A_200_ (μmol m^−2^ s^−1^)	22.4 ± 1.6^a^	14.7 ± 1.8^b^	22.9 ± 1.5^a^	11.8 ± 0.8^b^
*V*_cmax_ (μmol m^−2^ s^−1^)	65.3 ± 4.8^a^	29.0 ± 4.4^b^	72.8 ± 7.1^a^	19.9 ± 2.5^b^
*J*_max_ (μmol m^−2^ s^−1^)	93.8 ± 7.2^a^	62.3 ± 8.8^b^	97.5 ± 6.4^a^	46.9 ± 4.1^b^
Chlorophyll content (mg Chl m^−2^)	103.4 ± 1.3^a^	102.4 ± 0.5^a^	98.9 ± 0.6^a^	99.2 ± 0.3^a^

Measurements were made in plants grown under high (200 Pa CO_2_) and measured immediately (Day 0) or after 2 days in ambient CO_2_ (Day 2) under 1200 μmol PAR. Rates of CO_2_ exchange at reference intercellular CO_2_ concentration of ~200 Pa (A_200_) or ~40 Pa (A_40_), and maximum rates of Rubisco carboxylation (*V*_cmax_) and electron transport (*J*_max_) were calculated from a photosynthetic CO_2_ response curve. Means of *n* = 4 are shown with ± standard error. Significant differences within a measurement are indicated with different letters according to a two-way ANOVA with *p* < 0.05

Decreases in *V*_cmax_ in *plgg1*-*1* were explained by a reduction in the Rubisco activation state. Wild type and *plgg1*-*1* had identical Rubisco content expressed on a protein and leaf area basis (Table 4). While the Rubisco content was similar, *plgg1*-*1* had a significantly lower activation state as determined from in vitro activities. Chlorophyll content was similar between wild type and *plgg1*-*1* immediately following and after 2 days at ambient CO_2_ (Table 4).

**Table 4:** Rubisco content of wild type and plgg1-1

	Wild type	*plgg1*-*1*
Rubisco (μmol sites m^−2^)	8.8 ± 0.5^a^	8.4 ± 0.5^a^
Total protein (μg m^−2^)	6.6 ± 0.1^a^	7.2 ± 0.6^a^
Rubisco:protein (μmol sites μg^−1^)	1.3 ± 0.1^a^	1.2 ± 0.1^a^
Rubisco activation state (%)	82 ± 2.0^a^	69 ± 2.4^b^
Rubisco initial activity (μmol CO_2_ m^−2^ s^−1^)	21.3 ± 0.1^a^	21.0 ± 1.7^a^
Rubisco final activity (μmol CO_2_ m^−2^ s^−1^)	26.1 ± 0.8^a^	29.8 ± 1.9^a^

Rubisco content was measured in raw leaf extracts from the binding of ^14^CABP and total protein content quantified using the Bradford assay. Means of *n* = 3–7 are shown with ± standard error. Rubisco quantification, Rubisco activity assays, and chlorophyll extractions were performed on separate generations of plants. Significant differences between genotypes are indicated with different letters according to a Student's *t* test with *p* < 0.05

### *plgg1*-*1* has decreased photosynthetic activity in old, but not young, leaves following transition to ambient CO_2_

**Fig. 3:**
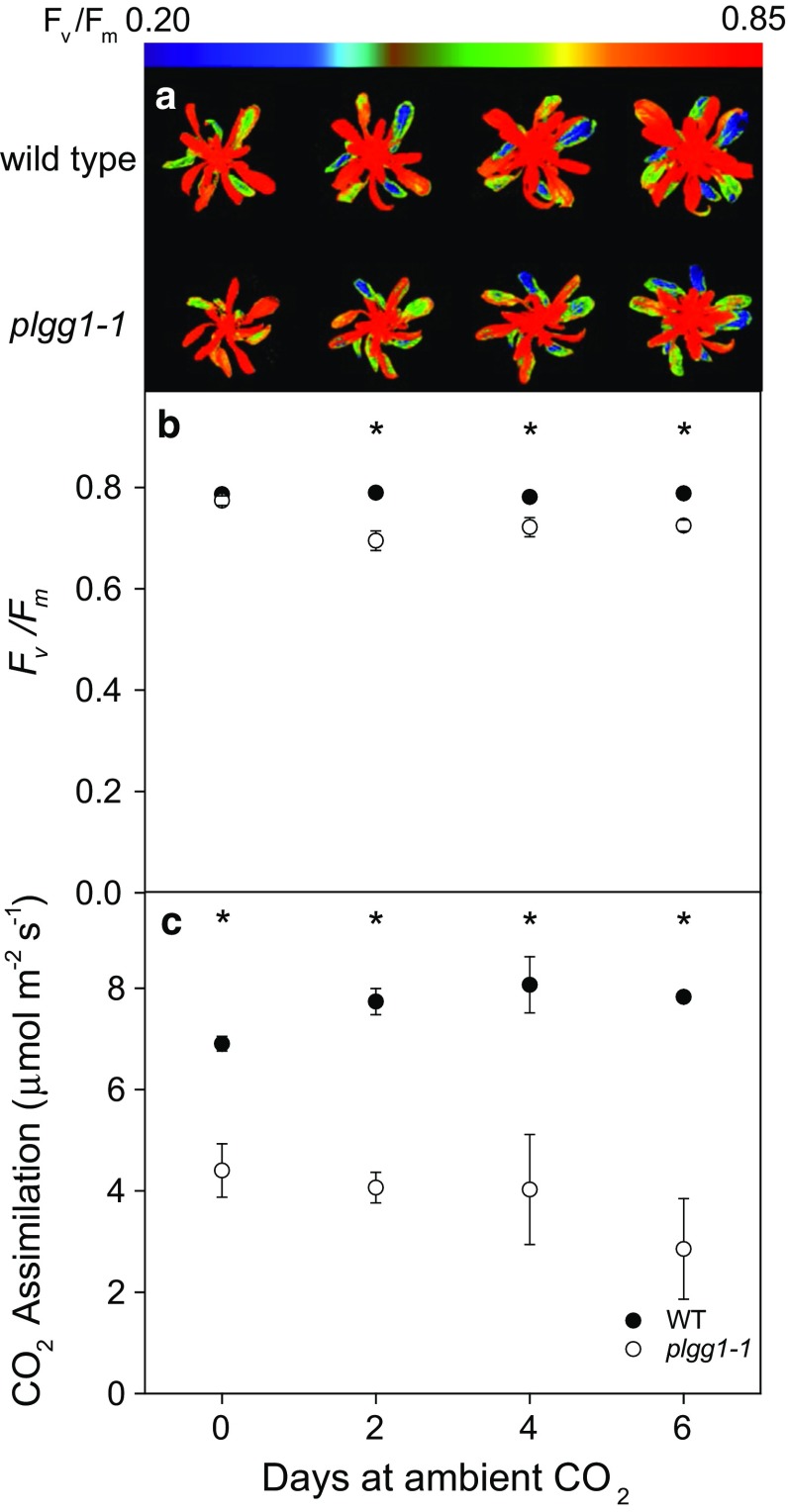
Representative color *F*_v_/*F*_m_ fluorescence images (**a**), *F*_v_/*F*_m_ of youngest fully expanded leaf (**b**), and CO_2_ assimilation rates (**c**) from wild type (WT) and *plgg*-*1* following transition from elevated (200 Pa) to ambient CO_2_. Plants were dark adapted for at least 20 min and measured with a saturating flash. Following imaging gas exchange was measured using a Li-Cor 6400XT on the healthiest available leaves as determined from chlorophyll fluorescence imaging. *Stars* indicate significant differences between genotypes on a given day as determined from a repeated measures two-way ANOVA with a Tukey post hoc test (*p* < 0.05). Bars represent means of *n* = 4 with ± standard error

Additional gas exchange and chlorophyll fluorescence imaging further highlighted the plasticity in the ability of *plgg1*-*1* to compensate for impaired glycolate/glycerate transport. Dark adapted *F*_v_/*F*_m_ values did not respond to transitions to ambient CO_2_ in wild type, but decreased by 10 % in *plgg1*-*1* after 2 days in ambient CO_2_ (Fig. 3a, b). In *plgg1*-*1*, *F*_v_/*F*_m_ decreased more in older leaves as compared to developing leaves (Fig. 3a) following exposure to ambient CO_2_ and did not increase following 2 days (Fig. 3b). This decrease in *F*_v_/*F*_m_ occurred in a patchy manner, with some regions of the older leaves showing a greater decrease than others making absolute quantification difficult. These areas of decreased *F*_v_/*F*_m_ in older leaves developed chlorotic lesions following 4 days of exposure to ambient CO_2_. Interestingly, young leaves continued to develop and plants set seeds under longer periods of exposure to ambient CO_2_. Initial experiments also indicated that *plgg1*-*1* could complete its lifecycle under ambient CO_2_ and produce viable seed under low light conditions. Net CO_2_ assimilation decreased in *plgg1*-*1* by 36 % on day 0 to 63 % on day 6 compared to wild type (Fig. 3c).

## Discussion

Despite lacking the key glycolate/glycerate exchange transporter of photorespiration, *plgg1*-*1* showed surprising physiological plasticity in maintaining photorespiratory flux in terms of photorespiratory recycling efficiency. Despite a modeled decrease in $\varPhi_{{{\text{CO}}_{2} }}$ with decreased photorespiratory efficiency, *plgg1*-*1* showed a similar $\varPhi_{{{\text{CO}}_{2} }}$ to wild type, even when measured near the CO_2_ compensation point for *plgg1*-*1* where rates of Rubisco oxygenation approach those of carboxylation (Fig. 1; Tables 1, 2). Furthermore, there was no significant increase in Γ^*^, suggesting that α is maintained in *plgg1*-*1* (Table 2; Eq. 1). While there was a statistically insignificantly higher Γ^*^ in *plgg1*-*1*, it was not as much higher as reported for *hprpmdh1pmdh2* (45 % increase, Cousins et al. 2011). Since both $\varPhi_{{{\text{CO}}_{2} }}$ and Γ^*^ are directly impacted by the efficiency of photorespiratory carbon recycling, these data provide two independent indications that *plgg1*-*1* is able to sustain near-normal operation of photorespiration. One caveat is that Rubisco activity is sensitive to irradiances and $\varPhi_{{{\text{CO}}_{2} }}$ and Γ^*^ are measured under sub-saturating light irradiances, so these findings are only necessary valid when absolute rates of photorespiration are low (Taylor and Terry 1984).

When light is saturating, a classic, all-be-it weak (i.e., *plgg1*-*1* plants can survive and grow slowly in ambient [CO_2_]), photorespiratory phenotype is found in *plgg1*-*1* with reduced photosynthetic rates that are partially rescued under low photorespiratory conditions in both light and A–C_i_ curves (Table 3; Fig. 1). Timm and Bauwe (2013) provide a framework for classifying the range of photorespiratory mutant phenotypes. Class I mutants are not rescued even under high (~2 kPa) CO_2_ partial pressures and require supplemental sucrose for growth. Class II mutants display the so-called “classic” photorespiratory phenotype and show a conditional lethality to ambient CO_2_ conditions. Class III mutants show retarded but viable growth at ambient CO_2_ that is compensated under elevated CO_2_, while class IV mutants show only slight response to ambient CO_2_ conditions. Using this classification scheme, we would classify *plgg1*-*1* as a class III photorespiratory mutant, placing it among other mutants such as those lacking glutamate–glyoxylate aminotransferase, glycine decarboxylase, and hydroxypyruvate decarboxylase. In addition to a phenotypic similarity to these mutants, *plgg1*-*1* appears similar to mutants lacking glycerate kinase given the relative decrease in *F*_v_/*F*_m_ and net CO_2_ assimilation as well as the increase in Γ following exposure to ambient CO_2_ (Timm et al. 2012). Perhaps coincidentally, the glycerate kinase mutation is only one reaction downstream of the glycerate transport that *PLGG1* mediates. These comparisons to other photorespiratory mutants are tentative however, since a valid comparison would need to be performed on all mutants in the same experiment.

Interestingly, the reductions in *plgg1*-*1* net photosynthetic rate can be explained by a 60 and 50 % decrease in *V*_cmax_ and *J*_max_, respectively, and not decreased photorespiratory recycling efficiency (Tables 2, 3). Decreases in both *V*_cmax_ and *J*_max_ also appear to explain the decrease in net assimilation in *plgg1*-*1* following multiple days under ambient CO_2_ (Table 3; Fig. 3). Since *V*_cmax_ and *J*_max_ are tied directly to changes in Rubisco activation state and photochemical efficiency of PSII, these decreases could be explained by decreases in the Rubisco activation state and increases in photoinhibition. Decreases in *V*_cmax_ could be at least partially driven by decreases in Rubisco activation state and not total Rubisco content (Table 4). The in vitro Rubisco activity assay did not appear sensitive enough to determine differences in the initial activity of Rubisco extracts (Table 3) but we feel that the combination of *V*_cmax_ with the same Rubisco content and lowered activation state are sufficient to attribute at least some the decrease in *V*_cmax_ to decreases in Rubisco activation state.

This possible decrease in Rubisco activation state may have been due to decreased Rubisco activation due to insufficient Rubisco activase activity in *plgg1*-*1,* which would explain the lowered CO_2_ assimilation at lower CO_2_ partial pressures. Decreased Rubisco activity would also explain why *plgg1*-*1* has a higher compensation point but similar Γ^*^ and *R*_d_, since the compensation point is sensitive to the ratio of day respiration to maximum Rubisco carboxylation rates (Eq. 2; Tables 2, 3). Similar decreases in Rubisco activity are found in *hprpmdh1pmdh2* and in rice plants inducibly expressing an antisense glycolate oxidase gene, suggesting that photorespiration may inhibit the C3 cycle via feedback mechanisms on Rubisco (Xu et al. 2009; Cousins et al. 2011). This observation is also an important consideration when interpreting changes to the compensation point in photorespiratory mutants, since increases can be explained by decreases in the maximum Rubisco carboxylation rate and not necessarily changes to photorespiratory efficiency (Timm et al. 2012).

The mechanism of photorespiratory-mediated deactivation of Rubisco is not clear, but could be mediated by photorespiratory metabolites directly. Early work examining isolated chloroplasts indicate that glycolate and glycerate builds up in the absence of peroxisomes and mitochondria (i.e., fully functioning photorespiratory cycle, Kearney and Tolbert 1962). It has been shown that Rubisco activation correlates with glyoxylate concentrations in vivo and in vitro (Chastain and Ogren 1989) and in intact, lysed and reconstituted chloroplasts (Campbell and Ogren 1990). Additionally, excess glyoxylate was shown to correlate with net photosynthesis in rice plants expressing down-regulated glycolate oxidase, although it is unclear why plants exhibiting decreased glycolate oxidase activity showed increases in glyoxylate concentrations (Lu et al. 2014). Interestingly, glyoxylate concentrations are *lower* in *plgg1*-*1* plants as compared to wild type immediately following transfer to ambient CO_2_, although glyoxylate increases somewhat (although insignificantly) in *plgg1*-*1* following 2 and 5 days at ambient CO_2_ (Pick et al. 2013). These seemingly contradictory findings suggest that the impact of glyoxylate on Rubisco activation state may be secondary, although clearly more work is needed to directly resolve and confirm this hypothesis. Glycolate is also greatly increased in *plgg1*-*1* (Pick et al. 2013), perhaps indicating it could exert a feedback control on Rubisco.

A similar decrease in *J*_max_ observed in *plgg1*-*1* (Table 3) is found in *hprpmdh1pmdh2,* which limits the maximum rate of net CO_2_ assimilation (Cousins et al. 2011). This decrease in the maximum rate of electron transport could result from increased photoinhibition as indicated by the decrease in dark adapted *F*_v_/*F*_m_ following exposure to ambient CO_2_, assuming that photosystem II centers were completely repaired during the dark adaptation period (Fig. 3a, b, Baker and Oxborough 2004). Increases in photoinhibition are found in other photorespiratory mutants and are attributed to the impaired repair of photoinhibition, but without more in-depth analysis, we cannot speculate further on the mechanism of our decreased *J*_max_ (Takahashi et al. 2007; Badger et al. 2009; Timm et al. 2012).

Decreases in *F*_v_/*F*_m_ were observed on the older leaves of the rosette, but not younger, expanding leaves (Fig. 3a). A similar pattern of decreased *F*_v_/*F*_m_ is observed in plants lacking the primary mitochondrial serine hydroxymethyltransferase and may indicate an acclimation response to ambient CO_2_ provided by a gene with overlapping function (Timm et al. 2012: Fig. 3b therein). Increased expression of a gene with overlapping transport activity could explain the plasticity of the *plgg1*-*1* phenotype in young but not older leaves, but more work would be needed to clarify this. The chlorophyll content of *plgg1*-*1* was similar to wild type (Table 3). This finding of similar chlorophyll contents differs somewhat from the finding that overexpression of a PLGG1 homolog in tomato, where overexpression of a *PLGG1* homolog resulted in decreased chlorophyll biosynthesis (*Solanum lycopersicum*, Kang et al. 2016).

Since the photorespiratory efficiencies of *plgg1*-*1* were unaffected under low rates of oxygenation, it is possible that the mutation is leaky, allowing normal photorespiratory operation compensated when fluxes were low. However, the *plgg1*-*1* line results from a T-DNA insertion in the first intron of *plgg1* and shows no *PLGG1* expression as determined from transcript abundance, and *plgg1* has no homologs in Arabidopsis (Pick et al. 2013), confirming that these mutants were complete knockouts. This suggests that either other genes, potentially un-described transporters, and/or glycolate diffusion across the chloroplast envelope are adequate to support moderate rates of photorespiration when PLGG1 is absent.

Longer periods of exposure to ambient CO_2_ led to decreased *F*_v_/*F*_m_ and chlorotic lesions in older leaves, similar to that observed previously (Pick et al. 2013). This observation indicates that the plasticity of photorespiration in *plgg1*-*1* cannot fully protect against leaf damage under these conditions, at least in mature leaves. It is possible that this leaf damage is secondary to the compensatory mechanism that preserves the efficiency of photorespiration immediately following transfer to ambient CO_2_, but not following longer term exposure. It is interesting to note that plants continued to develop under low CO_2_ and could even complete an entire life cycle under ambient CO_2_. These observations further suggest an alternative plastic mechanism for glycolate and glycerate transport in photorespiration. This mechanism could be an alternative low-affinity alternative transporter, pore, or simple diffusion through the chloroplast membrane. The *plgg1*-*1* shows increased glycolate and glycerate pools that increase with the number of days at ambient CO_2_ (Pick et al. 2013). While this metabolic evidence (and the additional transport activities) demonstrates *plgg1*-*1* is a glycolate/glycerate transporter, they do not exclude the possibility that alternative transport processes participate in photorespiration when substrate concentrations of glycolate and glycerate become high enough. The research presented here demonstrates that despite these elevated pools, the efficiency of photorespiration appears unaffected, although the absolute rate is changed based on changes in Rubisco activation state.

## Conclusions

Taken together, these observations lead us to hypothesize that *plgg1*-*1* compensates for impaired glycolate/glycerate transport by additional transport processes including un-described transporters and/or passive diffusion of glycolate across the chloroplast envelope as suggested previously (Pick et al. 2013). These alternative transport processes are adequate to maintain wild-type rates of net assimilation when net photorespiration is low as found under sub-saturating irradiance. As photorespiratory rates increase, net assimilation is decreased not by compromised photorespiratory efficiency, but by decreased Rubisco activity and electron transport. This mechanism may be specific to *plgg1*-*1*, but may also apply to other photorespiratory mutants or possibly when photorespiration is compromised in wild-type plants due to environmental factors such as increases in temperature, which could put similar pressures on photorespiratory metabolism.

## Electronic supplementary material

Below is the link to the electronic supplementary material.

Supplementary material 1 (DOCX 306 kb)
