# Supplementary material for: Physiological evidence for plasticity in glycolate/glycerate transport during photorespiration
Source: Photosynth Res. 2016 Jun 1;129:93–103. doi: 10.1007/s11120-016-0277-3 (PMC4906074; doi:10.1007/s11120-016-0277-3)
Supplement: Supplementary file 1 — Supplementary material 1 (DOCX 306 kb) [file 11120_2016_277_MOESM1_ESM.docx]

**Supplemental 1**

**Supplemental 1.** Individual common intercept measurements of Γ* and R_d_ in wild type and *plgg1-1*. Gas exchange was performed as outlined in the materials and methods. Rep and genotype is indicated at the top of each figure.

**Supplemental 2.** Photosynthetic CO_2_ response curves measured in wild type (WT) and *plgg1-1* immediately following transition to ambient CO_2_ (a) and after two days at ambient CO_2_ (b). Gas exchange was measured using a Li-Cor 6400XT on the healthiest available leaves as determined from chlorophyll fluorescence imaging. Bars represent means of n=4 with ± standard error.
